# Impact of clinical pathway on clinical outcomes in the management of COPD exacerbation

**DOI:** 10.1186/1471-2466-12-27

**Published:** 2012-06-22

**Authors:** Andrea Ban, Aniza Ismail, Roslan Harun, Azahirafairud Abdul Rahman, Saperi Sulung, Aljunid Syed Mohamed

**Affiliations:** 1 Department of Medicine, UKMMC, Kuala Lumpur, Malaysia; 2 Department of Community Health, UKMMC, Kuala Lumpur, Malaysia; 3 Case-mix Centre, UKMMC, Kuala Lumpur, Malaysia; 4 United Nations University-International Institute of Global Health (UNU-IIGH), Kuala Lumpur, Malaysia

**Keywords:** Chronic obstructive pulmonary disease, Clinical pathway, Clinical outcomes, Length of stay

## Abstract

**Background:**

Exacerbations, a leading cause of hospitalization in patients with chronic obstructive pulmonary disease (COPD), affect the quality of life and prognosis. Treatment recommendations as provided in the evidence-based guidelines are not consistently followed, partly due to absence of simplified task-oriented approach to care. In this study, we describe the development and implementation of a clinical pathway (CP) and evaluate its effectiveness in the management of COPD exacerbation.

**Methods:**

We developed a CP and evaluated its effectiveness in a non-randomized prospective study with historical controls on patients admitted for exacerbation of COPD to Universiti Kebangsaan Malaysia Medical Centre (UKMMC). Consecutive patients who were admitted between June 2009 and December 2010 were prospectively recruited into the CP group. Non-CP historical controls were obtained from case records of patients admitted between January 2008 and January 2009. Clinical outcomes were evaluated by comparing the length of stay (LOS), complication rates, readmissions, and mortality rates.

**Results:**

Ninety-five patients were recruited in the CP group and 98 patients were included in the non-CP historical group. Both groups were comparable with no significant differences in age, sex and severity of COPD (*p* = 0.641). For clinical outcome measures, patients in the CP group had shorter length of stay than the non-CP group (median (IQR): 5 (4–7) days versus 7 (7–9) days, *p* < 0.001) and 24.1% less complications (14.7% versus 38.8%, *p* < 0.001). We did not find any significant differences in readmission and mortality rates.

**Conclusion:**

The implementation of CP –reduced the length of stay and complication rates of patients hospitalized for acute exacerbation of COPD.

## Background

Chronic obstructive pulmonary disease (COPD) is characterised by progressive, partially reversible airflow obstruction and lung hyperinflation with significant extra-pulmonary manifestations and co-morbid conditions [1]. COPD is the fifth leading cause of death worldwide and the 12^th^ leading cause of disability [1,2]. In Asia, WHO estimates that the number of COPD cases will exceed by three times the total number of COPD cases for the rest of the world by the year 2020 [3]. In Malaysia, COPD ranked fifth in terms of disease burden [3] and its prevalence is projected to rise. This chronic condition is progressive in nature resulting in a steady decline in lung function. Its progression is accelerated by episodic exacerbations, typically requiring inpatient hospital care at increasing frequency as the disease progresses. A COPD exacerbation is characterized by an acute worsening of the patient’s baseline symptoms including dyspnoea, cough and/or sputum, which may require alteration in regular medication.

Exacerbations, a leading cause of hospitalization in patients with COPD, affect the quality of life and prognosis. All-cause mortality after hospitalization for COPD exacerbation is high up to 50% particularly in those requiring ventilator supports [4]. Patients with frequent COPD exacerbations have poorer quality of life and accelerated decline in lung functions. Suboptimal treatment of COPD exacerbations is a major concern. It increases health-care resources utilization and cost, and may sometimes cause detrimental effects to patients. Treatment recommendations as provided in the evidence-based guidelines are not consistently followed.

A large study in USA involving 70,000 patients admitted for acute COPD exacerbation found that only 30% patients received all the recommended treatment according to the established guidelines [5]. We have also previously shown that the compliance to our national guideline of COPD management was only 60% for the treatment of moderate to severe stable COPD [6]. Among the reasons for failure to adhere to the treatment recommendations include lack of awareness of the COPD guidelines particularly among the non-respiratory and primary care physicians, and absence of simplified task-oriented approach to care. Thus, there is a critical need to address this increasing gap between the guidelines and clinical practices, particularly in the hospitals of developing countries, by implementing and evaluating clinical care practices tailored to local settings. Clinical pathways can provide a link between establishing clinical guidelines and practicing them.

A clinical pathway (CP) - also known as a care pathway or multidisciplinary action plan (MAP) – is defined as an interventional plan for the mutual decision making and organization of care processes for a well-defined group of patients during a well-defined period [7]. The pathway outlines the main clinical interventions that are carried out in the hospital by a group of health care professionals responsible for the care of the patient. It incorporates locally agreed clinical standard practices as well as guidelines into a systematic approach for managing a specific group of patients with the aim of improving patient outcomes, promoting patient safety, increasing patient satisfaction, and optimizing the use of resources [7].

COPD presents a significant disease burden in Malaysia and is reflected in hospital admission rates for COPD exacerbations. The management of COPD admissions is guided by local standard practices and clinical guidelines established by the Ministry of Health of Malaysia. However there are inconsistencies between guidelines and their application in daily clinical practice. While existing clinical guidelines for COPD provide evidence-based and consensus-derived statements of optimal care for patients with COPD, such guidelines do not provide an organizational framework for managing patients within a hospital setting, resulting in inconsistent application of clinical plans and variable patient outcomes. COPD CP has been successfully implemented in developed countries [8] and previous studies suggest that its usage results in improved clinical outcomes [9,10]. The adoption of COPD CP in Asia, however, lags behind and evidence of its effectiveness in developing countries is less clear.

To date, there has been no published study on the effectiveness or impact of COPD clinical pathway development and implementation in Malaysia and also very few studies worldwide. In view of the positive outcomes reported in the literature in decreased LOS and readmission, we decided to examine the benefits and its effectiveness.

In this study, we report the development and implementation of a COPD CP in a teaching hospital in Malaysia, and evaluate its effectiveness by comparing the length of stay (LOS), complication rates, readmissions, and mortality between CP and non-CP COPD patients.

## Methods

This was a non-randomized, prospective study with historical controls in hospitalized patients with COPD in the Universiti Kebangsaan Malaysia Medical Centre (UKMMC). The study was carried out over a period of 31 months, from June 2008 to December 2010 starting from the development of COPD until patient recruitment. The CP was implemented in June 2009. All patients gave written informed consent and the study was approved by the Research and Ethics Committee of the Faculty of Medicine, Universiti Kebangsaan Malaysia (FF-154-2008).

The study population consisted of two groups which were the non-clinical pathway (non-CP group) and the clinical pathway group (CP group). The CP group was recruited prospectively from patients that were admitted with exacerbation of COPD between June 2009 and December 2010. For this study, an acute exacerbation of COPD (AECOPD) was defined as an acute worsening of dyspnoea, cough or change in quality or quantity of sputum requiring a change of regular medication. These symptoms should have been present for at least 3 days. We included adult patients aged 40 years and above with a diagnosis of exacerbation of COPD, and excluded patients with the following conditions: (i) congestive cardiac failure, (ii) long term oxygen therapy use (LTOT) ≥ 15 hours/day, (iii) underlying malignancy, (iv) active tuberculosis, (v) diffuse interstitial lung diseases, or (vi) pulmonary thromboembolic disease. Patients with LTOT were excluded as co-existing cor pulmonale would be a confounding factor that could prolong the length of hospitalization. Patients on LTOT represent a small subset of COPD patients in the advanced stages of their diseases. We chose to exclude these patients in our study.

For the non-CP group, COPD patients were recruited retrospectively from the medical records of patients admitted with a diagnosis of exacerbation of COPD between January 2008 and January 2009. Besides the clinical diagnosis by the attending physician, we required evidence of spirometry readings consistent with COPD: obstructive lung disease FEV1/FVC <70% with minimal reversibility (<12%), together with a significant smoking history of more than 10 pack-years. In our teaching hospital, the GOLD [2,11] and Malaysian Clinical Practice Guidelines (CPG) were available as references for the management of acute COPD exacerbation [1]. We limited the retrospective review of cases to 1- year (Jan 2008 to Jan 2009) prior to commencement of the prospective recruitment of the CP group (June 2009), so as to ensure the treatment regimes and access to pulmonary rehabilitation programs were comparable.

For comparison of both groups, we collected demographic data (age, gender, education, working status) as well as baseline characteristics (BMI, smoking status, COPD severity, and co-morbidities). Both groups were matched for age, gender and severity of COPD. COPD severity was classified based on the percentage predicted FEV1 in accordance to GOLD guidelines: stage 1 > = 80%; stage 2: 50–80%; stage 3: 30–49%; and stage 4 < 30%. (Table 1) [11]. In both groups all the data were documented and collected by medical officers who were in charged in the medical wards. 

**Table 1:** Stage of COPD and percentage predicted FEV1

**Stage of COPD**	**Percentage predicted FEV1**
Stage 1	> Or = 80%
Stage 2	50–80%
Stage 3	30–49
Stage 4	<30%

### Development and implementation of the COPD clinical pathway

The paper-based pathway for COPD was developed by Universiti Kebangsaan Malaysia Medical Centre (UKMMC) in collaboration with United Nations University International Institute for Global Health (UNU-IIGH). The development was conducted in series of workshops involving the Respiratory Unit of UKMMC, UNU-IGH, and the Department of Public Health. In the first phase, we determined the roles of the healthcare teams involved in the management of a COPD patient. This included the respiratory consultant and specialist, medical officers, the head nurse*,* staff nurses, pharmacist, dietitian, physiotherapist, and social worker. The clinical management of COPD was based on GOLD [12] and NICE [13] guidelines, as well as local best practices [1], but team members were allowed to deviate from the pathway depending on the clinical progress of the patient. We used this as a framework to decide on optimum days in the ward, the usage of oxygen therapy, antibiotic requirement and regimes, DVT prophylaxis and others. As a baseline, the expected length of stay was set at 5 days.

The COPD CP consisted of day-to-day assessment of the patients starting from day 1 until the day of discharge. These assessments included physiotherapy assessment, oxygen requirement assessment, treatment regimes; bronchodilator therapy corticosteroid and antibiotic requirement, deep venous thrombosis (DVT), fluid regime, blood tests and chest radiograph requirement were also listed in the assessment chart in the CP .

In the second phase, a pilot study was carried out to evaluate the feasibility of the COPD clinical pathway, and to modify the pathway accordingly Prior to starting the pilot study there was an official launch of the clinical pathway at the hospital level. This was officiated by the director of the UKMMC and attended by representatives from nurses, physiotherapists, pharmacists as well as medical doctors. A separate session to introduce the CP to the medical department was done as a continuous medical education (CME) session to the medical officers, house officers, nurses, pharmacists and physiotherapists who were based in the medical wards. The CME session was done to explain the clinical pathway in brief and outline the main roles to be played by each unit. In the third phase, we implemented the COPD CP in the medical wards in June 2009.

### Piloting of the care pathway in subsets of patients

Patients admitted to the medical wards with a diagnosis of exacerbation of COPD were screened within 24 hours of admission and managed according to the COPD CP if the patients fulfilled the inclusion/exclusion criteria. The CP worksheets were attached to the patient’s records, and to ensure compliance to the CP, we designated one study investigator (medical officer) to review the clinical pathway records daily and prompt the team to follow the specific goals set for each day. The study investigator performed daily ward rounds to enforce compliance on the usage of the pathway. The health care providers were given encouragement and reinforcement to implement the clinical pathway. Additional educational activities were also given to address specific issues raised during the implementation.

### Evaluation of COPD CP

The evaluation focused mainly on the care process and outcomes. The variables that were measured for evaluation were length of hospital stay (LOS), complications or morbidity, readmission and mortality. Confounders of LOS would include smoking status of patient, hospital acquired infections, and worsening of co-morbidities.

In this study readmission was defined as any unplanned hospital admission within 30 days after discharge. Patients were given the status of readmission only if they were admitted for clinical problems primarily related to the initial episode of COPD exacerbation. The interval of follow up was 30 days and this was done via a telephone call. Complications were defined as any disease or injury that developed during the treatment of a pre-existing disorder. It can vary from complication related to the pre-existing disease or non related.

### Statistical analysis

Sample size determination was calculated using Power and Sample Size Program (PS) package (version 3.0.43). Statistical comparisons were performed using Statistical Software Statistical Product and Services (SPSS) software package (version 19) using appropriate statistical tests at a significance level of 95%.

## Results

### Recruitment and baseline characteristics

The CP group was prospectively recruited from a total of 212 patients that were admitted with a diagnosis of COPD exacerbation between June 2009 and December 2010. Ninety-seven patients fulfilled the inclusion criteria and all were recruited in the CP group (46%); 95 patients completed the study (98%). For the historical non-CP control group, the medical case notes of 281 COPD patients admitted between January 2008 and January 2009 were reviewed. Only 160 patients had complete documentation of the case notes, and of these, 98 patients fulfilled the criteria and were included in the control group (61%).

The basic demographic distributions between the CP and non-CP groups were comparable and no significant differences were noted in age, gender, ethnicity, and working status (*p* > 0.05) (Table 2). We found that the non-CP group comprised of patients with lower levels of education compared to the CP group (*p* = 0.035).

**Table 2:** Baseline characteristics and demographic data between two groups

**Characteristic**	**Total N = 193**	**Non-Clinical Pathway (n = 98)**	**Clinical Pathway (n = 95)**	***P*value**
Age (years ± sd*)	68.48 ± 8.84	68.78 ± 8.45	68.18 ± 9.27	0.641^#^
Gender [n(%)]				
Male	168 (87.0)	82 (83.7)	86 (90.5)	0.156^ƛ^
Female	25 (13.0)	16 (16.3)	9 (9.5)	
Ethnicity [n(%)]				
Malay	51 (26.4)	25 (25.5)	26 (27.4)	0.353^£^
Chinese	110 (57.0)	61 (62.2)	49 (51.6)	
Indian	29 (15.0)	11 (11.2)	18 (18.9)	
Others	3 (1.6)	1 (1.1)	2 (2.1)	
Level of education [n(%)]				
No formal education	31 (16.1)	18 (18.4)	13 (13.7)	0.035^ƛ^
Primary	94 (48.7)	54 (55.1)	40 (42.1)	
Secondary	68 (35.2)	26 (26.5)	42 (44.2)	
Working status [n(%)]				
Work(non-professional)	37 (19.2)	15 (15.5)	22 (23.2)	0.166^ƛ^
Not working	156 (80.8)	83 (84.7)	73 (76.8)	
BMI in kg/m^2^ (range)	24.4 (20.9–27.2)	24.6 (21.5–27.5)	23.8 (19.1–27.0)	0.382^α^
BMI category (%)				
Underweight (<18.5)	24 (14.4)	5 (6.9)	19 (20.0)	0.029^£^
Normal (18.5 – 22.9)	42 (25.1)	23 (31.9)	19 (20.0)	
Overweight (23 – 27.4)	67 (40.1)	26 (36.1)	41 (43.2)	
Obese (>27.4)	32 (20.4)	18 (25.0)	16 (16.8)	
Smoking status [n(%)]				
Current smoker	22 (11.4)	17 (17.3)	5 (5.3)	0.008^ƛ^
Ex-smoker	171 (88.6)	81 (82.7)	90 (94.7)	
Stage of disease [n(%)]				
Stage 2	7 (4.0)	4 (4.8)	3 (3.4)	0.924^£^
Stage 3	46 (26.6)	22 (26.2)	24 (27.0)	
Stage 4	120 (69.4)	58 (69.0)	62 (69.7)	
Co Morbidity [n(%)]				
NO	83 (43.0)	38 (38.8)	45 (47.4)	0.228^ƛ^
YES	110 (57.0)	60 (61.2)	50 (52.6)	
Types of Co morbidity[n(%)]				
Hypertension	91 (47.2)	49 (50.0)	42 (44.2)	0.421^ƛ^
Diabetes Mellitus	37 (19.2)	18 (18.4)	19 (20.0)	0.773^ƛ^
Ischemic heart disease	52 (26.9)	28 (28.6)	24 (25.3)	0.605^ƛ^
Dyslipidemia	48 (24.9)	26 (26.5)	22 (23.2)	0.588^ƛ^
Chronic kidney disease	10 (5.2)	5 (5.1)	5 (5.3)	0.607^£^

* sd = standard deviation α = Mann Whitney *U* test, # = independent *t* test, ^ƛ^ = Pearson Chi-Square test, £ = Fisher’s Exact test, Significant level *P* < 0.05.

Next we examined baseline characteristics of BMI, smoking status, COPD stage, and co-morbidities (Table 2). No differences were noted in the COPD stage of disease and presence of co-morbidities (*p* > 0.05) between the two groups. However, the CP group had significantly more underweight patients compared to the non-CP group (20% vs 6.9%) (*p* = 0.029). When we examined the smoking status, all patients had a history of smoking in both groups, but the non-CP group had a higher percentage of current smokers compared to CP group (17.3% vs 5.3%) (*p* = 0.008).

### Evaluation of clinical outcomes

#### Length of stay (LOS)

The overall length of stay ranged from 2 to 13 days. The median LOS of the overall COPD population was 6 days (means 6.59 ± 2.15 days). The median LOS in non-CP group was 7 days (mean = 7.31 ± 2.75 days) whilst LOS in CP group was 5 days (5.83 ± 1.92 days) (*p* < 0.001) (Table 3).

**Table 3:** Comparison of non-CP and CP group with average length of stay (ALOS)

**Variable**	**Non-Clinical Pathway (n = 98)**	**Clinical Pathway (n = 95)**	***P*value**
Average length of stay in days (LOS)	7.31 ± 2.75	5.83 ± 1.92	<0.001^#^
*Median LOS in days	7 (7–9)	5 (4–7)	<0.001^ψ^

# = independent *t* test, *Median IQR (25^th^-75^th^), ψ = Mann Whitney *U* test, Significant level *P* <0.05.

#### Complication rates

The CP group had less number of complications (38 patients, 38.8%) compared to the non-CP group (14 patients, 14.7%), and this difference was statistically significant (*p* < 0.001) (Table 4).

**Table 4:** Type of complications and readmission between groups

**Characteristic**	**Non-Clinical Pathway (n = 38)**	**Clinical Pathway*P*Value (n = 14)**
Type of complications (n)		
Acute respiratory failure	9	5
Hospital Acquired Pneumonia	7	3
Acute Coronary Syndrome	5	0
Congestive Cardiac Failure/	2	2
Fluid overload		
Acute Renal Failure	3	2
Thromboplebitis/cellulitis	3	1
Upper gastrointestinal bleeding	1	0
Urinary tract infection	2	0
Others	6	2
Readmission (< 30 days)		
No	76 (77.6)	73 (76.8) *0.907
Yes	22 (22.4)	22 (23.2)

*^ƛ^ = Pearson Chi-Square test, Significant level *P* value <0.05.

The commonest complication was acute respiratory failure leading to noninvasive positive pressure ventilation (NIPPV) or invasive positive pressure ventilation (IPPV) in both groups followed by hospital acquired pneumonia (HAP). Other complications were acute coronary syndrome (ACS), congestive cardiac failure (CCF) or fluid overload, acute renal failure (ARF) thrombophlebitis and cellulitis upper gastrointestinal bleeding (UGIB) urinary tract infection (UTI) and others, which include uncontrolled hypertensive and diabetes, acute gouty arthritis and stroke. (Table 4).

#### Number of readmissions

There were a total of 44 patients readmitted within 30 days of discharge. There was no difference between in the number of readmissions between the groups: 22 (22.4%) in non-CP group and 22 (23.2%) in CP group (*p* > 0.05).

#### Mortality rates

There were two deaths in the CP group (2.1%) and none in the non-CP group (0%). This difference was not significant (*P* = 0.24).

## Discussion

In this study, we developed and implemented a clinical pathway for the management of COPD exacerbations, and compared the outcomes (LOS, complication rate, readmission rate and mortality) of patients managed with the COPD clinical pathway with patients previously managed using the standard care guidelines.

As a baseline for comparison, we used historical controls from patients that were most recently managed using standard care guidelines one year prior to implementation of the clinical pathway. This control group was comparable to the patients that were prospectively recruited in the clinical pathway group: both groups had similar access to drugs, pulmonary rehabilitation, and smoking cessation clinic sessions; and their demographic profiles were similar. We did not find significant differences in the age, gender, and ethnicity between the groups.

The median BMI of the study population was 24.4 (20.9–27.2) which is classified as overweight following the Malaysian obesity guidelines (MOH 2004). Sixty one percent of the patients were overweight and obese, while 25.1% had normal BMI and 14.4% were underweight. Rampal et al. 2007 showed that the overall national prevalence of obesity in Malaysia was 11.7% [14], however, we noted that they classified the cut-off point of obesity as BMI ≥ 30 kg/m^2^. We classified obesity as a BMI ≥ 27.5 kg/m^2^ as recommended by the Malaysian clinical guidelines in management of obesity 2004[15].

A total of 171(88.6%) subjects were ex-smokers and 22(11.4%) were still actively smoking. There were more active smokers (17%) in the non-CP group compared to CP group (5.3%). This may have contributed to the differences in LOS between the groups. It is unfortunate that we failed to match this factor between the groups. The CP group had their smoking history taken with the knowledge that they were participating in a clinical trial. There is a possibility that they may have under reported their years of smoking. The historical controls from case notes had smoking history taken and verified over a few entries. This may have been more reflective of an accurate number of pack years and smoking status. However there was no significant association between smoking history and length of stay in the two groups. The study also found no significant association between smoking history and complication. When we removed the 22 current smokers and reanalyzed the data, the length of stay between the CP and non-CP group was still significant (*p* < 0.001).

In terms of severity of COPD, majority of the patients admitted for COPD exacerbation had moderate to severe stage of disease. This is in keeping with the role of UKMMC as a tertiary referral centre. Another possible reason is most COPD patients approach their doctor at a later stage of the disease.

As COPD patients are generally elderly, comorbidities are expected to be higher. More than 50% of COPD patients in this study had at least one comorbid disease. The most common comorbid disease was hypertension. A large study by Mannino et al. (2008) reported that lung function impairment was associated with more comorbid disease [16]. The risk of hospitalization and mortality were increased with comorbid disease, in which the mortality was worse in patients with impaired lung function [16]. Prevalence of diabetes was found to be increased in COPD where 15% of the patients with COPD admitted to the hospital had diabetes mellitus [16]. This result was almost similar to the prevalence of diabetes in our COPD population (19.2%). Antonelli et al. (1997) found that selected comorbids including chronic renal failure and myocardial infarction or ischemia were able to predict mortality in patients with advanced COPD [17].

This study confirmed our hypothesis that usage of CP in the management of acute exacerbation of COPD decreased the hospital length of stay (LOS). We found a significant reduction in LOS of the CP group as compared to the non-CP group. The overall median LOS of COPD patients was 6 days and ranged between 2 to 13 days. The median LOS in non-CP group was 7 days whilst the LOS in CP group was 5 days. A Study by Celis et al. (2004), which utilized the COPD CP, also showed a significant reduction of mean hospital stay of 13.21 days in the control group and 10.24 days in the CP group [18]. A similar study by Santamaria showed shortening of 0.89 days (13.2%) of hospital stay in the pathway group; however, this was not statistically significant [9]. This was a prospective unblinded study in a single unit and the non-CP group had more co-morbidities. This could have contributed to the LOS being not significant. A retrospective study by Celis et al. (2004) also did demonstrate a significant decrease in LOS [18], possibly due to the retrospective nature of the study.

For other respiratory disease CPs, Bailey et al. (1998) showed that the use of CP in asthma also caused reduction in the LOS [19]. This reduction in LOS was also noted after implementation of pneumonia clinical pathway [20,21].

There is insufficient data to indicate the optimal duration of hospitalization in patients with COPD exacerbation. A study on necessary length of hospital stay for COPD done by Mushlin et al. (1991) found that 6.9 days was considered averaged LOS [22]. Our overall (both non-CP and CP groups) mean LOS was 6.56 days. We developed a 5-day COPD CP based on the recommended average use of antibiotic (3 to 7 days) from GOLD guidelines. Our CP was similar to COPD CP from the Grey Bruce Health Network in this aspect (approximately 5 days target). This study showed that CP can help in reducing length of stay and indirectly reducing cost of care.

The non-CP group had significantly more complications than the CP group (38 vs. 13). This is consistent with the study by Santamaria et al. (2004) which also showed fewer complications in the CP group [9]. Acute respiratory failure (14 cases) was the most common complication seen which is similar to the study by Santamaria et al. (2004) [9]. COPD patients are prone to develop type II respiratory failure with CO2 retention which would lead to mechanical ventilation support.

The number of readmissions was similar in both groups. We found no significant difference in the number of unplanned readmissions between the two groups. The main reason for readmission in this study was shortness of breath. The CP group also had longer readmission interval after discharge. Our findings were consistent with the study by Santamaria et al. [9]. The earliest unplanned readmission was four days in the CP group and one day in the non-CP group. There is an on-going study by the EUROPEAN Quality of Care Pathways Study on COPD (EQCP-COPD) looking at readmission rates as a primary outcome.

There was no significant association in mortality between the non-CP and CP groups. Our finding was similar to Santamaria et al. study [9]. There were two deaths in the CP group. Both patients were in stage IV COPD. One was a 70-year old male ex-smoker who had hypertension and ischaemic heart disease. The second subject was an 81-year old male who had no comorbidities. Both deaths were associated with COPD exacerbation.

We encountered minimal initial resistance to the clinical pathway. However, this was not a major issue. This lack of acceptance resulted in some deviation from the clinical pathway. Several batches of medical officers and house officers were rotated into the medical unit during the duration of this study. Information on the COPD clinical pathway may not be well disseminated. Our briefing sessions should have been more regular to improve the compliance and adherence to the CP. In our study it was not possible to screen admission during long weekends or public holidays. We designated a single investigator to review and prompt the doctors. This problem can be improved by appointing case managers in each ward. Due to the retrospective nature of case control section of this study, there were a number of incomplete data documentation and recording.

This study on COPD clinical pathway in Malaysia showed a decrease in LOS and complications. We are currently evaluating the effectiveness of the CP using this current data from a cost analysis point of view. This will be published upon completion of analysis. We continue to improve our COPD clinical pathway by studying the variances. We will use these to tailor the current CP to reflect our resources available to us.

## Conclusions

In conclusion we have developed and implemented the first clinical pathway for inpatient management of acute COPD exacerbation in Malaysia. Our study suggests that the usage of CP in the management of COPD exacerbation decreases the hospital length of stay and complication rates. The clinical pathway integrates the routine assessment of a COPD patient. This can help identify patients at risk of pseudomonal infections and streamline their antibiotic therapy. In our study doctors were prompted to change antibiotics as well as steroid therapy to the oral form from day 2 of admission. Doctors were also prompted to switch to regular bronchodilator therapy MDI from day 2 of admission. There was a clear discharge plan listed in the clinical pathway. These factors may have contributed to a shorter length of stay in hospital.

We recommend its usage in other public hospitals in Malaysia to improve the efficiency and quality of care in COPD.

## Competing interests

The authors declare that we have no competing interest.

## Authors’ contributions

ARA = study design, data collection, data analysis and interpretation, drafting and revising manuscript. AYLB = study design, design of CP, data analysis and interpretation, revising manuscript. IA = study design, data collection, data analysis and interpretation, drafting and revising manuscript. HR = study design, design of the CP, revising manuscript. BSS = introduced the idea of CP in Malaysia, revising manuscript. SMA = introduced the idea of CP in Malaysia, revising manuscript. All authors read and approved the final manuscript.

## Pre-publication history

The pre-publication history for this paper can be accessed here:

http://www.biomedcentral.com/1471-2466/12/27/prepub
